# Application of Bone Marrow Mesenchymal Stem Cells Effectively Eliminates Endotoxemia to Protect Rat from Acute Liver Failure Induced by Thioacetamide

**DOI:** 10.1007/s13770-021-00421-5

**Published:** 2022-02-05

**Authors:** Ting Jiang, Geng Xia, Bo Yang, Hong-wei Zhang, Yue-shan Yin, Cheng-wei Tang, Jin-hui Yang

**Affiliations:** 1 Department of Gastroenterology, The Second Affiliated Hospital of Kunming Medical University, 374 Dianmian Avenue, Kunming, 650106 Yunnan China; 2 Institute of Medical Biology, Chinese Academy of Medical Sciences, 935 Jiaoling Road, Kunming, 650031 Yunnan China; 3 Department of Gastroenterology, West China Hospital, Sichuan University, 37 Guoxue lane, Chengdu, 610044 Sichuan China

**Keywords:** Bone marrow mesenchymal stem cells, Acute liver failure, Endotoxemia, Differentiation, PI3K

## Abstract

**Background:**

Endotoxemia is related to worse clinical outcomes in acute liver failure (ALF), but its management remains unsatisfactory. In this study, we aimed to assess whether the application of bone marrow mesenchymal stem cells (BMSCs) could eliminate endotoxemia and protect rats against ALF induced by thioacetamide (TAA).

**Methods:**

BMSCs were isolated from rats and identified by the specific morphology, differentiation potential, and surface markers. The optimal dose of TAA for this study was explored and TAA-induced ALF rats were randomized to three groups: the normal control group (Saline), ALF group (TAA + Saline), and BMSCs-treated group (TAA + BMSCs). The intestinal migration and differentiation of BMSCs was tracked *in vivo*, and intestinal permeability, endotoxin and inflammatory cytokines, histology, and mortality were analyzed. Moreover, we added the inhibitor of the PI3K/AKT/mTOR signaling pathway into the co-culture system of BMSCs with enterocytes and then performed CK and Villin expression experiments to assess the role of PI3K/AKT/mTOR signal pathway in the intestinal differentiation of BMSCs.

**Results:**

BMSCs migrated to the intestinal injury sites and differentiated into enterocytes, intestinal permeability was decreased compared with the ALF group. The higher expression of endotoxin and inflammatory cytokines were reversed after BMSCs transplantation in rats with ALF. Mortality and intestinal lesion were significantly decreased. Blocking the PI3K/AKT/mTOR signal pathway inhibited BMSCs’ intestinal differentiation *in vitro*.

**Conclusion:**

BMSCs can eliminate endotoxemia and reduce mortality in rats with ALF, and the PI3K/AKT/mTOR signal pathway is involved in intestinal differentiation. BMSCs transplantation could be a potential candidate for the treatment of endotoxemia in ALF.

## Introduction

Acute liver failure (ALF) is characterized by a sudden development of liver dysfunction and rapid hepatocellular necrosis, may progress to hepatic coma even death due to various etiologies, including drug toxicity, hepatic ischemia, immune-mediated attack, and viral hepatitis such as hepatitis B and hepatitis C [1, 2]. Endotoxemia represents the severe stage of ALF and is associated with mortality and heavy economic and social burden [3]. Reasons for endotoxemia in ALF include intestinal and hepatic factors, which also interact with each other [4]. Although liver transplantation is considered a curative treatment, the disparity between demand and supply and severe postoperative complications limit the applicability [5, 6]. Therefore, prevention and treatment of endotoxemia are particularly important in ALF clinical practices.

Endotoxin is mainly produced by gram-negative bacteria which overgrow in the disordered intestinal environment. The disordered intestinal environment in ALF is usually associated with liver dysfunction, congestion of portal vein, and growth of pathogenetic microorganisms [7]. First and foremost, severe liver dysfunction can result in the accumulation of toxic metabolites in circulation, such as putrescine, histamine, spermine, and spermidine, which are not only harmful to brain cells called the hepatic coma, but also a great challenge to enterocytes. A recent study shows that spermine and spermidine have a dose-dependent cytotoxic effect on intestinal cells grown in culture [8]. It has been reported that histamine was toxic for HT29 intestinal cells and induced cell apoptosis in a comparative analysis [9]. These data strongly suggest that liver function is essential to maintain a healthy intestinal environment. Meanwhile, the increased portal vein pressure caused by liver failure leads to intestinal congestion, which enhances the concentrations of toxic metabolites in the gut and causes intestinal mucosal barrier injury. Furthermore, the gut barrier damage could promote intestinal bacteria-endotoxin translocation and endotoxemia, which can increase the severity of the liver disease. Obviously, it is a vicious circle between liver and gut, the high mortality will be the last result. The effective treatment and prevention of endotoxemia can break the vicious circle.

Mesenchymal stem cells (MSCs) attract a lot of attention owing to the excellent ability of self-renewal and differentiation into various tissues. Furthermore, compared with embryonic (ESCs) and induced pluripotent stem cells (iPSCs), MSCs are easily isolated from bone marrow called the bone marrow mesenchymal stem cells (BMSCs) and characterized without the limitation of ethical problems, the way of cell retention and immunological rejection [10, 11]. Recent studies have described the applications of BMSCs for liver diseases. BMSCs could migrate to the injured liver and regenerate in the mice receiving partial hepatectomy (PH) and even improve the alleviation of liver fibrosis [12–14]. Moreover, the application of BMSCs has demonstrated the potential for the alleviation of liver injury and inflammation by inhibiting the activation of inflammatory factors [15, 16]. However, studies on BMSCs therapy for endotoxemia in ALF were scarce.

The potential of BMSCs therapy largely depends on their migration, proliferation, and differentiation, regulated by multiple signaling pathways and factors, including the Wnt signal transduction pathway, oxidative stress, transforming growth factor beta (TGF-β) pathway, and phosphoinositide kinase-3 (PI3K)/protein kinase B (AKT) signal pathway [17–20]. Among them, the classical PI3K/AKT signaling pathway is crucial, affecting the migration, proliferation, and intestinal differentiation of BMSCs [21]. The activated AKT initiates downstream mammalian target of rapamycin (mTOR)/p70 S6K1 signaling resulting in the expression of genes that control protein synthesis, cell growth, and proliferation [22]. Therefore, we speculate that the PI3K/AKT/mTOR signaling pathway may induce intestinal differentiation and trigger downstream target genes.

In this study, we aimed to explore the effects of BMSCs on ALF rats by repairing the intestinal mucosal barrier and eliminating endotoxemia. Moreover, we used LY294002 to inhibit the PI3K/AKT/mTOR signal pathway *in vitro* and then observed the changes in intestinal differentiation of BMSCs.

## Materials and methods

### Preparation of rat BMSCs

#### Isolation of rat BMSCs

BMSCs were isolated from SD rats (half male and female, 6–8 weeks old, 200 ± 10 g) obtained from the experimental animal center of Kunming Medical University as described previously [23]. BMSCs were maintained in high-glucose Dulbecco’s modified Eagle’s medium (DMEM; cat. RNBJ9730; Sigma-Aldrich, St. Louis, MO, USA) containing 15% fetal bovine serum (FBS; Cat. 10,099,141; Thermo Fisher Scientific, Inc., Waltham, MA, USA), 100 U/ml penicillin and 100 μg/ml streptomycin (Cat. SV30010; Hyclone, Thermo Fisher Scientific, Inc., Logan, UT, USA) in a humidified incubator maintained at 37 °C under 5% CO_2_.

#### *In vitro* adipogenic differentiation and oil red O staining

The cells used for adipogenic differentiation were cultured with the adipogenic medium (Cat. 70,811; Saiye Bio, Guangzhou, China) for 16 days. Then, cells were stained with oil red O (Cat. 91,412; Saiye Bio). In brief, cells were washed with phosphate buffer saline (PBS; Cat. SH30256.02; Hyclone, Thermo Fisher Scientific, Inc.) and fixed with 4% paraformaldehyde (Cat. 51,829; Saiye Bio) for 30 min. After washing with PBS three times, cells were incubated with oil red O working solution for 30 min. Red color lipid droplets could be checked microscopically.

#### Flow cytometry

Cells harvested from SD rats were collected, centrifuged, counted, and adjusted to 1 × 10^6^ cells/ml/tube. Cells were washed by PBS twice and resuspended in 100 μl PBS, then incubated with anti-CD34 (Cat. no. sc-7324; Santa Cruz Biotechnology, Inc., Santa Cruz, CA, USA), anti-CD45 (Cat. no. 202207; Biolegend, San Diego, CA, USA), anti-CD90 (Cat. no. 202526; Biolegend) and anti-CD29 (Cat. no. ab36219; Abcam, Cambridge, UK) antibodies at room temperature in dark for half an hour. A FACS flow cytometer (CytoFLEX; Beckman Coulter, Inc., Brea, CA, USA) was used.

#### Cell cycle assays

Cells were washed twice with cold PBS and made into a 1 × 10^6^/ml single-cell suspension, fixed with cold 70% ethanol at 4 °C overnight. After washing with 1 ml PBS once, cells were incubated with 500 μl PBS containing 50 μg/ml propidium iodide (PI; Cat. ab14083; Abcam), 100 μg/ml RNase A (Cat. 9001-99-4; Sigma-Aldrich), and 0.2% Triton X-100 (Cat. 9002-93-1; Solarbio Life Science, Beijing, China) at 4 °C in dark for 30 min and analyzed using a flow cytometer.

### *In vivo* study

#### The ALF rat models and the application of BMSCs

In this study, ALF was induced by thioacetamide (TAA; Cat. no. HY-N1131, MedChemExpress, Shanghai, China) as previously described [24–26]. All rats (half male and female, 6–8 weeks old, 200 ± 10 g) were maintained under a specific pathogen-free condition with a 12-h light/dark cycle and free access to food and tap water at the Animal Facilities of the Kunming Medical University. To get an optimal animal model for this study, we divided the rats into groups to explore the best TAA dose. The rats were randomly allocated to 4 groups (n = 25 each group). Group 1 (control group) received saline via gavage for two days, and group 2–4 (ALF groups) received different doses (200 mg, 300 mg, 400 mg/Kg/day; 2 days) of TAA via gavage. Then, after confirming the appropriate dose of TAA, we explored the therapeutic effect of BMSCs on rats with ALF. Rats were divided into 3 groups (n = 20 each group): saline (gavage/caudal vein), TAA (gavage) + saline (caudal vein), and TAA (gavage) + BMSCs (1 × 10^6^ cells, caudal vein). All rats were sacrificed by CO_2_ asphyxiation for examinations.

#### Histology

Samples (livers and intestines) from rats were fixed in 4% paraformaldehyde, dehydrated, and embedded in paraffin. Sections (4 μm) were stained with hematoxylin and eosin (H&E; Cat. 57,390; Saiye Bio). Images (× 100 magnification) were taken from each sample, and histopathological evaluation of the entire section was performed in a blinded manner. The injury of livers and intestines was scored using the modified histopathologic score described previously [27–29]. These criteria for the histological score were shown in Table 1.

**Table 1:** Histological criteria for the assessment of liver and intestine damage

Score	Liver	Intestine
0	No damage	No damage
1	Minimal congestion and vacuolization, single-cell necrosis	Mild focal epithelial edema and necrosis
2	Mild congestion and vacuolization, < 30% necrosis	Moderate diffuse swelling and necrosis of the villi
3	Moderate congestion and vacuolization, 30–60% necrosis	Severe necrosis with evidence of neutrophil infiltration in the submucosa
4	Severe congestion and vacuolization, > 60% necrosis	Major widespread necrosis with massive neutrophil infiltration and evidence of hemorrhage

#### *In vivo* assessment of intestinal barrier permeability

Animals were gavaged with FITC-labeled dextran (6 mg/100 g body weight; Cat. 60,842-46-8, Sigma). Food and water were withdrawn 4 h before the FITC-labeled dextran gavage. Then plasma was collected 4 h after gavage. The fluorescence intensity of the plasma was measured using a plate reader (Cytation1; Biotek, Winusky, VT, USA). The FITC-labeled dextran concentrations in plasma were determined by the standard curve.

#### RNA isolation and quantitative reverse transcription-polymerase chain reaction (qRT-PCR)

Total RNA was isolated from the intestinal tissues using the RNAiso kit (Cat. 9109; TaKaRa, Kyoto, Japan), according to the manufacturer’s protocol. cDNA was generated using the HiScript II 1st Strand cDNA Synthesis Kit (Cat. R211-01; Vazyme Biotech Co., Nanjing, China). Primers were listed in Table 2 and ChamQTM Universal SYBR qPCR master mix was obtained from the company (Cat. Q711-02; Vazyme Biotech Co.). Gene expression was calculated relative to glyceraldehyde-3- phosphate dehydrogenase (GAPDH).

**Table 2:** Forward and reverse primers sequences of genes

Gene	Forward primers sequences (5′-3′)	Reverse primers sequences (5′-3′)
Occludin	TGAAAGTCCACCTCCTTACAGA	CCGGATAAAAAGAGTACGCTGG
JAM A	GGCAAGGGT TCGGTGTACA	GGCAACTTGACAGAGTCGTTCTC
GAPDH	CCTTCATTGACCTCAACTACATG	CTTCTCCATGGTGGTGAAGAC

JAM A= junctional adhesion molecule A, GAPDH= glyceraldehyde-3- phosphate dehydrogenase

#### Tracking BMSCs *in vivo*

BMSCs were incubated with BrdU labeled solution (final concentration 10 μmol/L; Cat. no. 6813, Cell Signaling Technology, Inc., Boston, MA USA) for 48 h and then washed five times with serum-free DMEM medium, digested with 0.25% trypsin (Cat. T4049; Sigma-Aldrich), and made a cell suspension (1 × 10^7^/ml) for transplantation. The intestines of rats were obtained and fixed in 4% paraformaldehyde and embedded in paraffin. Sections were cut taking a standard procedure for immunofluorescence staining (Cat. no. 4408/2369/4414; Cell Signaling Technology, Inc.). BMSCs cells (BrdU^+^ cells) and intestinal cells differentiated from BMSCs (BrdU^+^Villin^+^ cells) in intestinal mucosa could be observed under the microscope.

#### Determination of diamine oxidase (DAO) and endotoxin

The concentration of DAO in plasma was measured using a commercial kit (Cat. no. ab241004; Abcam) according to the manufacturer’s instructions. The DAO activity could be measured when plasma was incubated with enzyme mix for 1 h at 37 °C. The endotoxin in plasma was assayed by the endotoxin kit (Cat. no. A39552; Thermo Fisher Scientific, Inc.).

#### Enzyme-linked immunosorbent assay (ELISA)

The levels of interleukin-6 (IL-6) and tumor necrosis factor-alpha (TNF-α) in serum were determined according to the manufacturer’s instructions of the ELISA kits (IL-6; Cat. no. BMS625; Thermo Fisher Scientific, Inc.) (TNF-α; Cat. no. KRC3011; Thermo Fisher Scientific, Inc.).

### Intestinal differentiation and transwell co-culture system

Transwell co-culture system (Cat. PIEP30R48; Merck Millipore, Darmstadt, Hesse-Darmstadt, Germany) was used for intestinal differentiation of BMSCs *in vitro*. Briefly, the lower chamber was loaded with BMSCs (1 × 10^6^ cells/well) and the upper insert was loaded with epithelial cells of the small intestinal crypt in rats (IEC-6; 1 × 10^6^ cells/well) which were obtained from Kunming Cell Bank of Chinese Academy of Sciences (Cat. no. KCB200720YJ). The induction system was placed in an incubator with 5% CO_2_ at 37 °C for 10 days. To inhibit the PI3K/AKT/mTOR signaling pathway, the inhibitor LY294002 (20 μM; Cat. no. ab120243; Abcam) was added to the co-culture system on the first day.

### Immunofluorescence

Cells were fixed with PBS containing 4% paraformaldehyde for 30 min at room temperature and permeabilized with 0.2% TritonX-100 for 10 min, then blocked in 2% BSA (Cat. 67,858; Saiye Bio) for 30 min. After that, the cells were incubated with a specific primary antibody and then with a secondary antibody, and finally stained with 4′,6-diamidino-2-phenylindole (DAPI; Cat. 57,881; Saiye Bio). The cell slides were observed under an Olympus fluorescence microscope. The antibodies we used were: cytokeratin (CK), villin, and secondary antibody. (Cat. no. ab68260/ab201989/ab150113; Abcam). Scoring was based on the previously proposed method [30].

### Western blot analysis

Cells and intestinal tissues were homogenized and lysed for 30 min on ice. The lysates were centrifuged at 12,000 rpm for 10 min. Proteins isolated from samples were running on 10% sodium dodecyl sulfate–polyacrylamide gel electrophoresis (SDS-PAGE) gels and then transferred to a polyvinylidene fluoride (PVDF) membrane (Cat. 3,010,040,001; Millipore, Billerica, MA, USA). Blots were blocked for 1 h at room temperature with 5% nonfat dried milk. After washing in tris-buffered saline Tween-20 (TBST; Cat. no.56711; Saiye Bio), the membranes were immunoblotted with the following antibodies: anti-IL-6, anti-TNF-α, anti-AKT, anti-mTOR, anti-CK, anti-Villin, anti-β actin (Cat. no. ab9770/ab66579/ab8805/ab32028/ab115293/ab109516/ab8227; Abcam). The blots were then incubated with the corresponding horseradish peroxidase-conjugated IgG (Cat. no. ab7090 and ab7125; Abcam), and the signals were detected using a chemiluminescence reagent.

### Statistical analysis

Data are presented as mean ± standard deviation (SD). Differences between groups were analyzed by Student *t*-test or Mann–Whitney *U* test when appropriate. Survival analyses were described by Kaplan–Meier method and compared by the Log-rank test. The data were processed using GraphPad Prism 7 (GraphPad Software, San Diego, CA, USA) and SPSS 23.0 (IBM Corp., Armonk, NY, USA). A *p* value of < 0.05 was considered statistically significant.

## Results

### Identification of BMSCs from rats

**Fig. 1:**
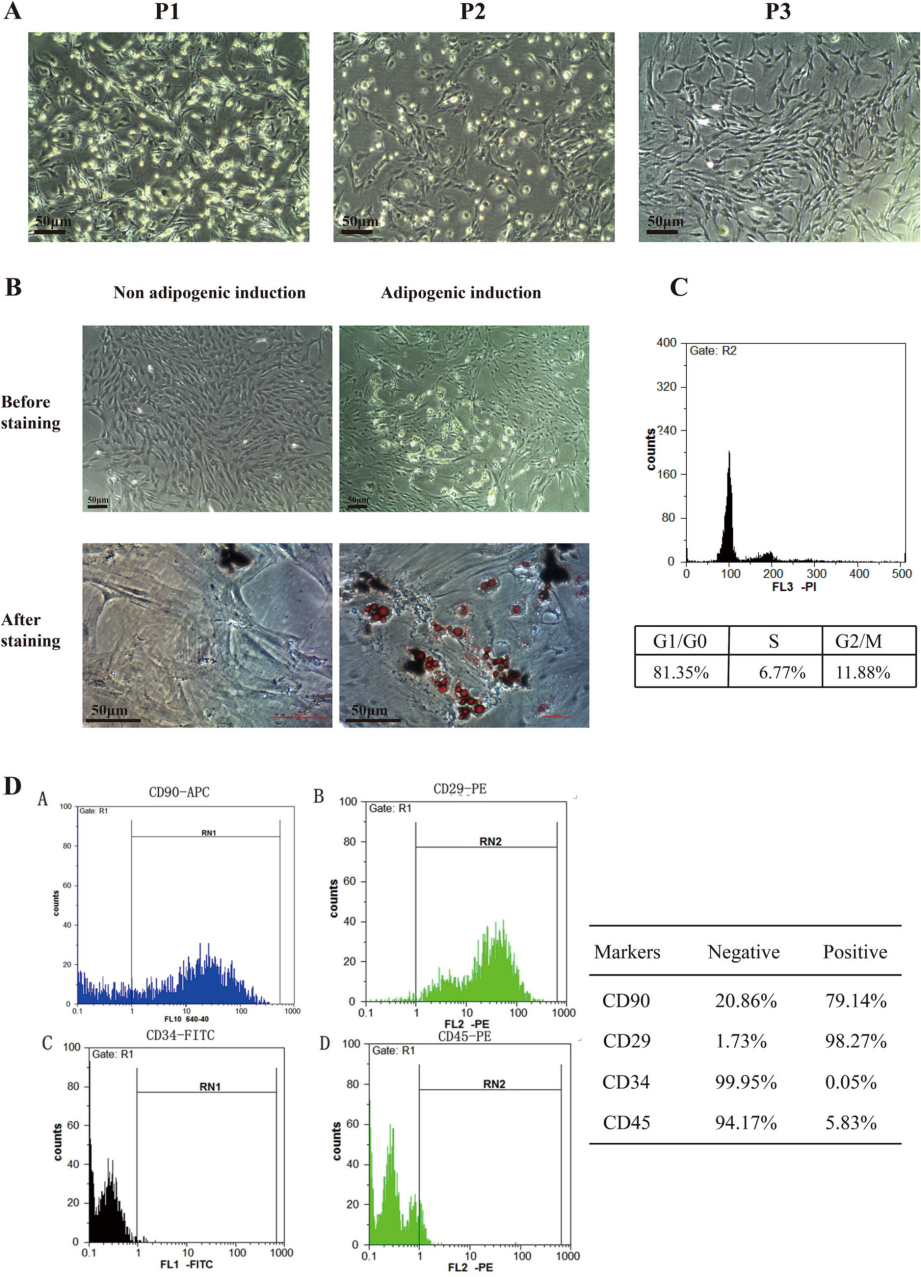
Characteristics of the isolated cells. **A** The morphology of passage 1~3 (P1/P2/P3) cells from rats. **B** The adipogenic induction and oil red O staining. **C** The analysis of the cell cycle of the isolated cells by flow cytometry. **D** The expression of surface markers of the isolated cells; Typical hematologic lineage CD29 and CD90 were expressed in cells, while CD45 and CD34 were not. Scale bars, 50 μm

The bone marrow of SD rats was used for isolation of the BMSCs. When the cells were cultured to the third passage, most of the cells were arranged in a whirlpool type and adhered to the wall of dishes, the shape of the cells was typically fibroblast-like or spindle-shaped (Fig. 1A). To demonstrate the pluripotency of the isolated cells, we induced adipogenic differentiation. Oil red O staining showed that droplets of fat were in the cells after the adipogenic induction compared with the control group (Fig. 1B). The specific surface markers of stem cells were detected by flow cytometry. We could notice the high expressions of CD90 (79.14%) and CD29 (98.27%) but low levels of CD45 (5.83%) and CD34 (0.05%) (Fig. 1). Taken together, these isolated cells could be identified as BMSCs. In addition, to ensure the follow-up experiments, we tested the cell cycle of BMSCs. As shown in Fig. 1C, these cells were in a state with a good ability for proliferation, that meets our experimental requirements.

### Effects of different doses of TAA on rats for ALF

**Fig. 2:**
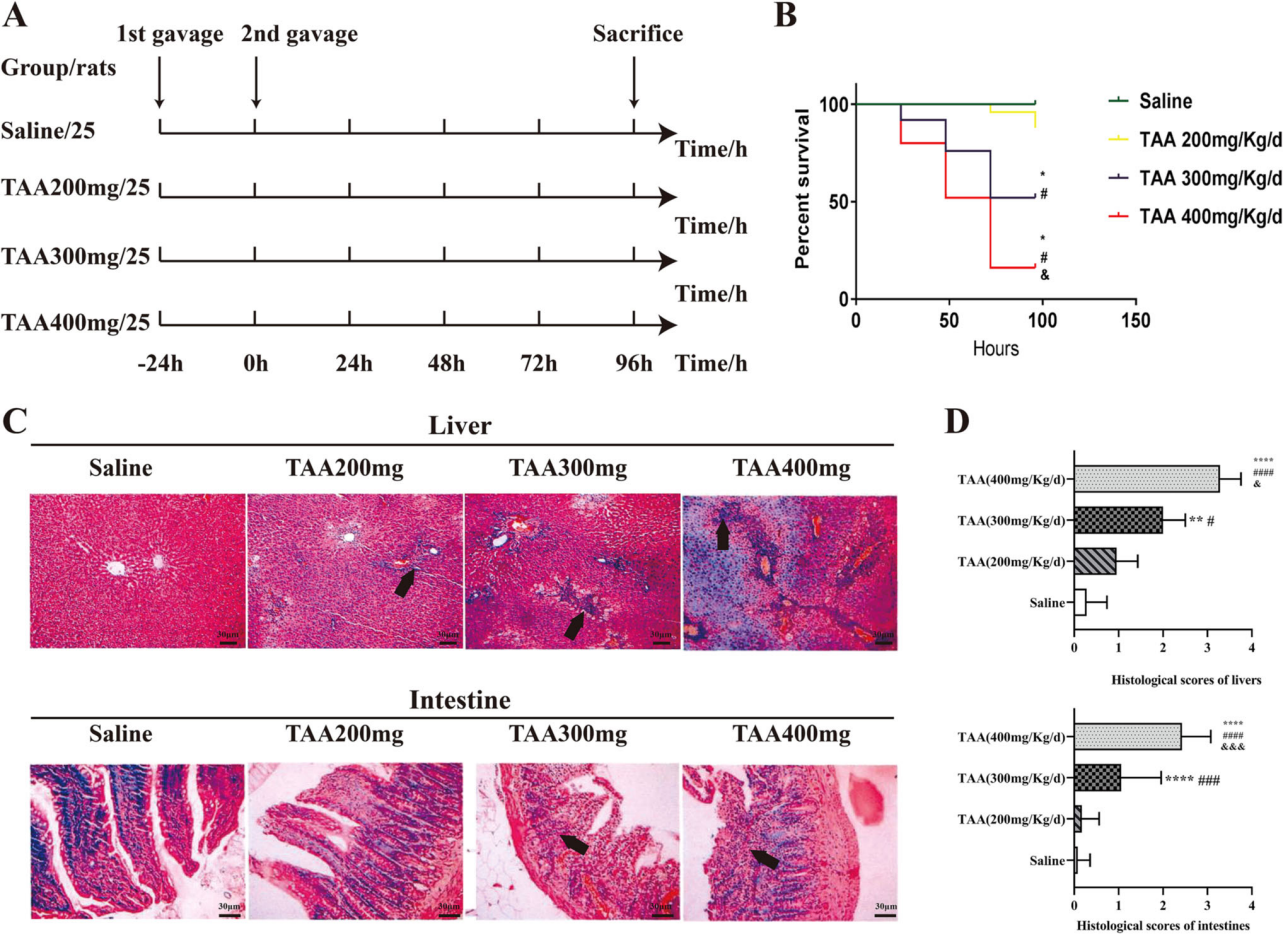
Animal models of acute liver failure induced by TAA. **A** Grouping and administration of TAA. **B** Survival analysis. * *p* < 0.05 vs. saline; # *p* < 0.05 vs. TAA 200 mg/Kg/d; & *p* < 0.05 vs. TAA 300 mg/Kg/d. **C** H&E staining; Necrosis of hepatocytes (upper black arrows), swelling and necrosis of the villi (lower black arrows); Scale bars, 30 μm. **D** Histology analysis of four groups. ** *p* < 0.01, **** *p* < 0.0001 vs. saline; # *p* < 0.05, ### *p* < 0.001, #### *p* < 0.0001 vs. TAA 200 mg/Kg/d; & *p* < 0.05, &&& *p* < 0.001 vs. TAA 300 mg/Kg/d. TAA, thioacetamide

Intestine lesions based on the ALF and appropriate survival rate were two bases for this study. To get an optimal ALF animal model, we divided the rats into four groups to explore the effects of different doses of TAA (Fig. 2). Both the group of TAA 300 mg/Kg/day and the group of TAA 400 mg/Kg/day showed typical hepatoenteropathology of ALF (Fig. 2C, D), while the group of TAA 400 mg/Kg/day had higher mortality (Fig. 2B), which was difficult to guarantee the follow-up experiments. Therefore, for this study, the dosage of TAA 300 mg/Kg/day was adopted.

### BMSCs migrated to the site of the injured intestine and affected intestinal permeability

**Fig. 3:**
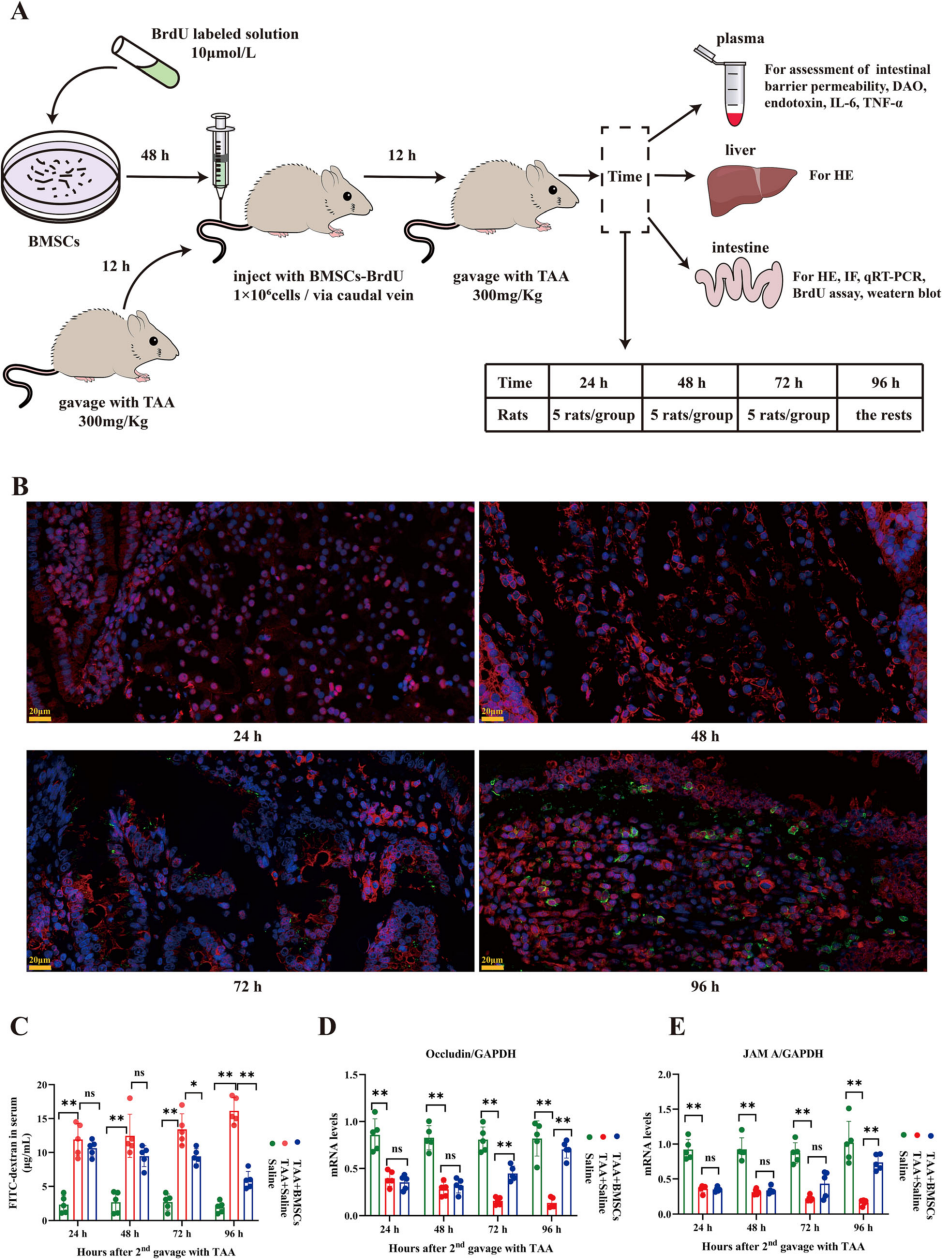
Intestinal migration of BMSCs and decreased intestinal permeability. **A** Flow chart of animal experiments. **B** Colocalization of EdU-labeled BMSCs and Villin was observed using immunofluorescence; DAPI-blue, BMSCs-BrdU-green; Villin-red Scale bars, 20 μm. **C** Fluorescence readings in plasma 4 h after gavage with FITC-dextran from groups. **D**, **E** Intestine mRNA levels of Occludin and JAM A; Values are normalized with GAPDH. ns *p* > 0.05, * *p* < 0.05, ** *p* < 0.01. TAA, thioacetamide; DAO, diamine oxidase; JAM A, junctional adhesion molecule A, GAPDH, glyceraldehyde-3- phosphate dehydrogenase

The general experimental procedure of this animal study is shown in Fig. 3A. About 72–96 h after injection of the BrdU-labeled BMSCs, the results showed the transplanted BMSCs had migrated into the injured intestine and differentiated into enterocytes (BrdU^+^Villin^+^ cells) (Fig. 3B). We next measured the consequence of BMSCs on intestinal permeability *in vivo*. BMSCs decreased intestinal permeability, as reflected by reduced plasma dextran concentrations (72 h, *p* = 0.02; 96 h, *p* = 0.01) (Fig. 3C). Consistently, these intestinal alterations were associated with higher mRNA expression of occludin (72 h, *p* = 0.01; 96 h, *p* = 0.008) and JAM A (96 h, *p* = 0.007) in the BMSCs-treated group (Fig. 3D, E). These results suggested that BMSCs migrated to the site of the injured intestine and differentiated into enterocytes, resulting in the reconstruction of the intestinal epithelial barrier.

### The administration of BMSCs ameliorated endotoxemia and reduced the mortality of rats with TAA-induced ALF

**Fig. 4:**
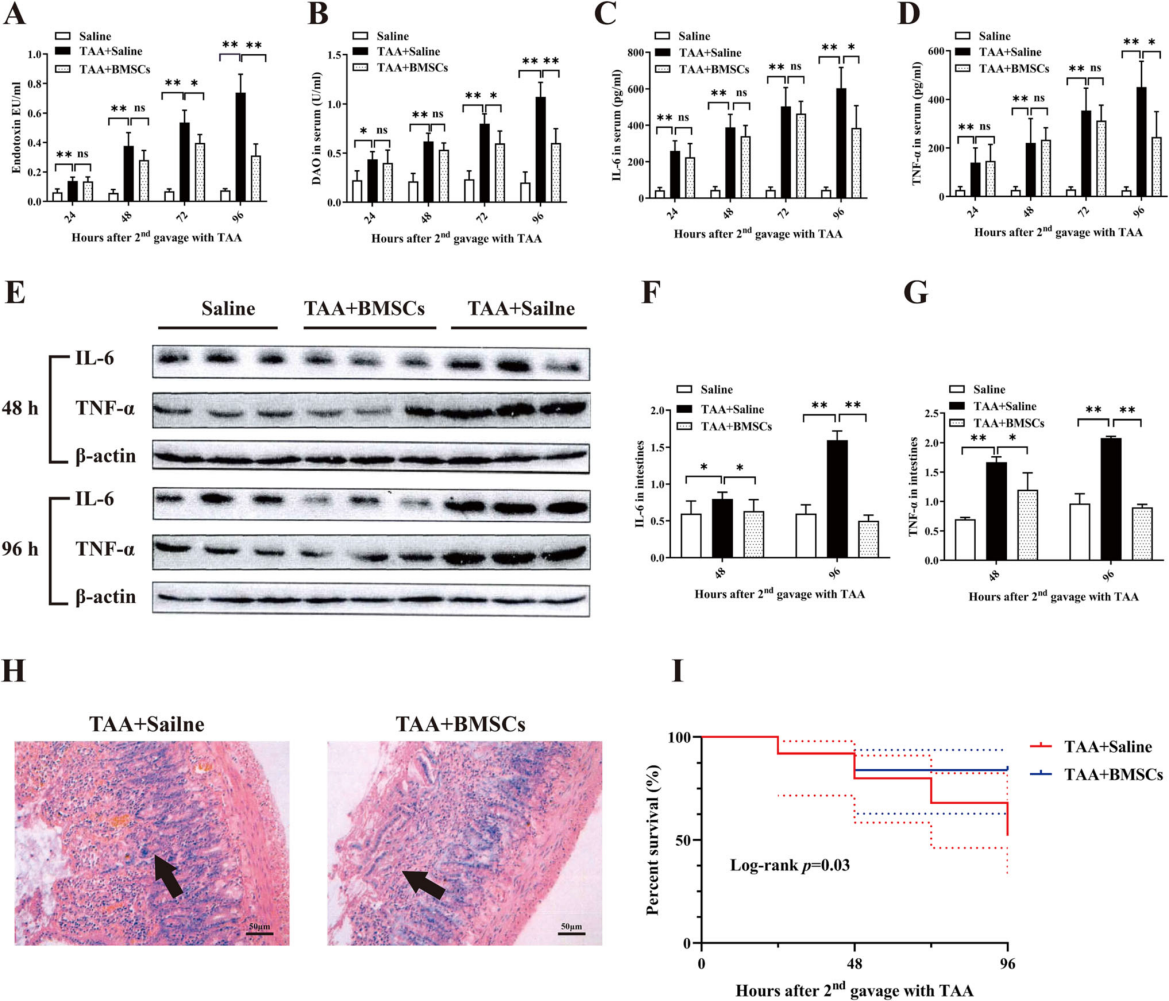
BMSCs administration eliminated endotoxemia and reduced mortality in the acute liver failure *in vivo*. **A** Endotoxin levels in serum. **B** DAO levels in serum. **C** IL-6 levels in serum. **D** TNF-α levels in serum. **E** IL-6 and TNF-α protein expressions in intestines measured by Western blotting. **F** and **G** The expressions of IL-6 and TNF-α decreased greatly in the BMSCs group than in the control group. **H** H&E staining of rat intestines; welling and necrosis of the villi were milder in the BMSCs group (black arrows); Scale bars 50 μm. **I** Survival analysis of groups; *p* = 0.03. ns *p* > 0.05, * *p* < 0.05, ** *p* < 0.01. TAA, thioacetamide; DAO, diamine oxidase; IL-6, interleukin-6; TNF-α, tumor necrosis factor-alpha

When compared with TAA + saline animals, the endotoxemia from rats administrated with BMSCs has been greatly alleviated, as shown by reductions in both the endotoxin (72 h, *p* = 0.02; 96 h, *p* = 0.007) and the DAO (72 h, *p* = 0.03; 96 h, *p* = 0.008) in serum (Fig. 4A, B). As IL-6 and TNF-α were important proinflammatory cytokines in ALF, we measured the expressions after the administration of BMSCs. Significant decreases in serum IL-6 (96 h, *p* = 0.03) and TNF-α (96 h, *p* = 0.03) were observed after BMSCs transplantation (Fig. 4C, D). A trend for the decrease in intestinal proinflammatory cytokine was also noticed by western blot (Fig. 4E). BMSCs led to significant reductions in IL-6 (48 h, *p* = 0.02; 96 h, *p* = 0.007) and TNF-α (48 h, *p* = 0.04; 96 h, *p* = 0.006) in intestines (Fig. 4F, G). From the morphological point of view, when the rats were treated with BMSCs, the intestine architecture was well preserved and the intestine was completely prevented from the edema and necrosis induced by TAA (Fig. 4H). Moreover, total mortality was higher (*p* = 0.03) in TAA + saline rats (12 of 25 deaths, 48% mortality) than in TAA + BMSCs rats (4 of 25 deaths, 16% mortality), the administration of BMSCs reduced mortality among ALF rats (Fig. 4I).

### PI3K/AKT/mTOR signal pathway was involved in the intestinal differentiation of BMSCs *in vitro*

**Fig. 5:**
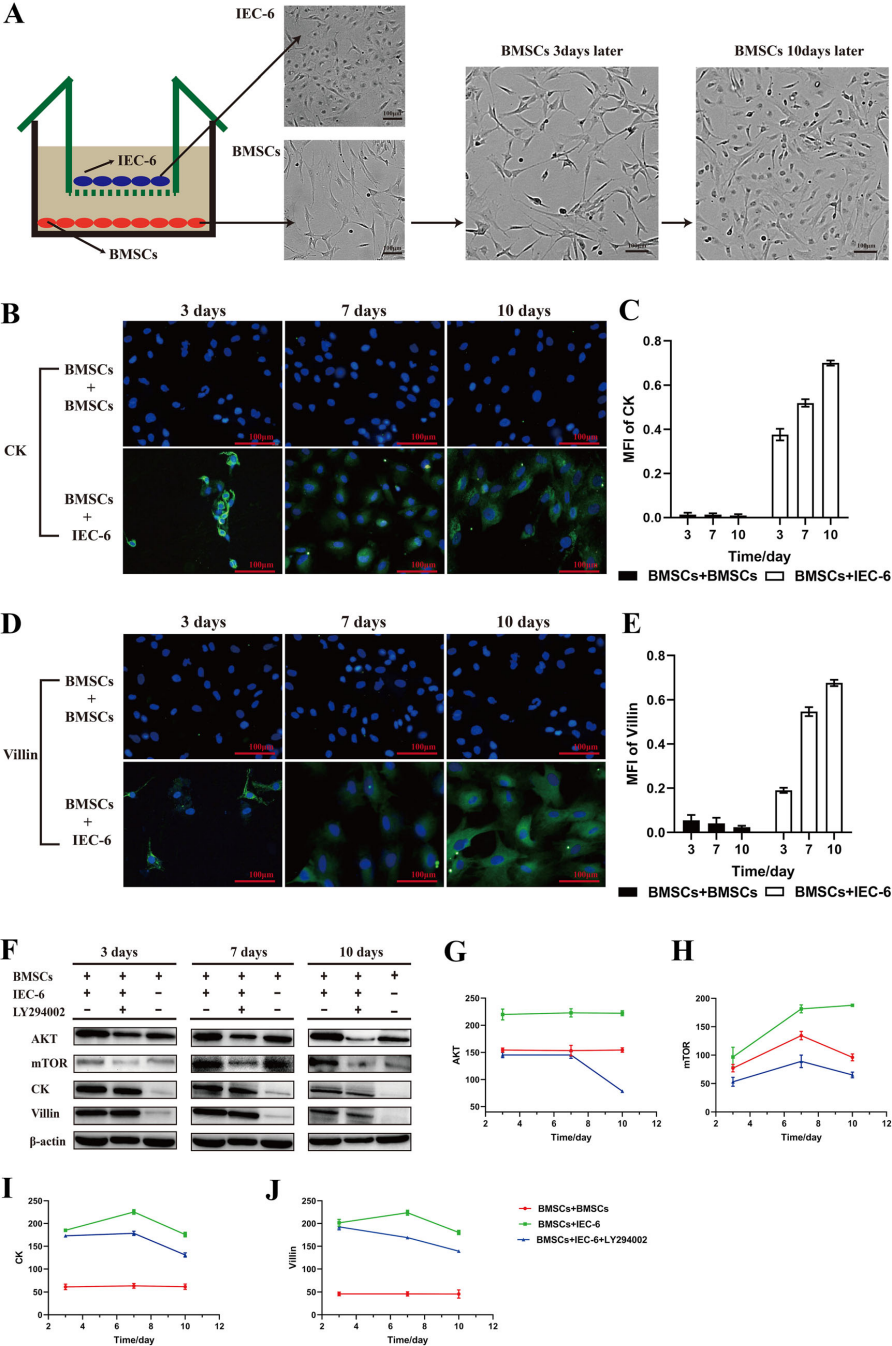
PI3K/AKT/mTOR signaling pathway was involved in the intestinal differentiation of BMSCs in vitro. **A** The spindle-shaped BMSCs morphology changed to a polygonal, round-like IEC-6 morphology in transwell co-culture system. **B**–**E** Immunofluorescence was performed to exhibit CK and Villin expression in the co-culture system. Scale bars 100 μm; DAPI-blue, CK-green, Villin-green. The levels of CK and Villin were increased in the BMSCs + IEC-6 group than in the BMSCs + BMSCs group. **F** AKT, mTOR, CK, and Villin protein expressions in different co-culture systems measured by Western blotting. **G**–**J** The expressions of CK and Villin decreased greatly when blocking the PI3K/AKT/mTOR signaling pathway. PI3K, phosphoinositide kinase-3; AKT, protein kinase B; mTOR, mammalian target of rapamycin

The above results in this study showed that BMSCs could migrate to the intestinal injury sites and differentiate into enterocytes, resulting in the reconstruction of the intestinal epithelial barrier and following reductions of endotoxin and mortality. Furthermore, we performed the transwell co-culture system to investigate whether the PI3K/AKT/mTOR signaling pathway is associated with the intestinal differentiation of BMSCs. Our results showed that the spindle-shaped BMSCs morphology changed to a polygonal, round-like IEC-6 morphology after the co-culture (Fig. 5). In addition, CK and Villin, two specific molecules of enterocytes, were found to be highly expressed in BMSCs which were co cultured with IEC-6 (Fig. 5B, D). Statistical analysis showed that after a 10-day co-culture, the levels of CK and Villin in BMSCs with IEC-6 were more than those in BMSCs without IEC-6 (Fig. 5C, E). Furthermore, the effect of the PI3K/AKT/mTOR signaling pathway in BMSCs’ intestinal differentiation was investigated by western blot (Fig. 5F). The results showed significant decreases in CK and Villin in the LY294002 group compared with the control groups (Fig. 5G–J). These data suggested that the PI3K/AKT/mTOR signaling pathway was involved in the intestinal differentiation of BMSCs *in vitro*.

## Discussion

The severe clinical outcomes of patients with ALF are closely related to the occurrence of hepatic coma, which is usually caused by endotoxemia. Indeed, endotoxemia is observed in 75–93.3% of ALF and severe hepatitis [31]. The common treatments such as antibiotics, artificial liver support systems, and nutritional support are still unsatisfactory [32]. Currently, with an inspiring development in stem cell technology, cell therapy has attracted a lot of interest in liver diseases. In an open-label randomized controlled study, the administration of allogeneic BMSCs reduced serum total bilirubin (TBIL) and model for end-stage liver disease (MELD) scores in 56 patients with acute-on-chronic liver failure (ACLF) [33]. Some studies proved that MSCs could differentiate into hepatocyte-like cells (HLCs) *in vivo*, considering as one of the mechanisms of MSCs in the treatment of liver failure [34, 35]. While some experiments have suggested that MSCs cannot differentiate into HLCs after engrafting even if the administration can improve survival, reduce alanine aminotransferase (ALT), and relieve liver injury [36]. These findings demonstrate that the therapeutic mechanism of MSCs in liver failure is not just dependent on the liver engrafting of exogenous stem cells and liver repairing.

It is worth mentioning that some inflammatory factors including IL-6, TNF-α, and CD8^+^T cells, which are induced by bacteria and endotoxins (lipopolysaccharide, LPS) from the gut, are significantly decreased after the BMSCs application in liver failure [37]. Previous studies have indicated that the gut plays a significant role in endotoxemia based on liver failure. The level of plasma endotoxin in the colectomized rats had no difference from that in the control group, the contents of inflammatory factors and fatality were much lower in these ALF rats. Therefore, we investigated the role of BMSCs in the gut and endotoxemia while on ALF. The results suggested that the levels of plasma endotoxin and DAO were remarkably decreased in the BMSCs group compared to the TAA + saline groups. Also, the concentrations of IL-6 and TNF-α decreased markedly, resulting in an important increase in survival rate. Histology with intestines from groups showed that a better intestinal mucosal barrier was observed after the BMSCs application. Moreover, the levels of IL-6 and TNF-α in the intestine were strongly reduced in rats with BMSCs. These findings suggested that BMSCs were indeed effective in the treatment of ALF by eliminating endotoxemia and rebuilding the intestinal epithelial barrier.

The intestinal differentiation of BMSCs was thought to be involved in many therapeutic studies on intestinal diseases. A recent phase III randomized controlled trial showed fistula healing in patients with Crohn's perianal fistulae treated via MSCs, and the reprogramming that allows MSCs to differentiate and migrate tissues was discussed [38]. Consequently, the animal experiment showed the activated inflammatory pathway in inflammatory bowel disease could be suppressed by stem cell transplantation to differentiate into intestinal epithelium [39]. In this study, we displayed the migration and intestinal differentiation of transplanted BMSCs in the injured intestinal tissues and the effects of intestinal repair, which was consistent with previous studies. we also carried out the co-culture system of BMSCs and IEC-6 *in vitro* using transwell and blocked the PI3K/AKT/mTOR signal pathway. The data demonstrated that the blocking of the PI3K/AKT/mTOR signal pathway using LY294002 could inhibit intestinal differentiation, suggesting the PI3K/AKT/mTOR signal pathway may regulate intestinal differentiation and intestinal repair in ALF. However, the present study only investigated the PI3K/AKT signaling pathway *in vitro*. Further experiments *in vivo* are required to confirm the results of this study. Meanwhile, some previous studies have stated that the therapeutic effects of BMSCs were based on its released trophic and immunomodulatory factors such as TGF-β and interleukin 10 (IL-10) [40]. Also, BMSCs can inhibit cytotoxic CD8^+^ T lymphocyte (CTL) and NK cells through intercellular contact and paracrine factors including TGF-β and IL-10. Thus, we can not rule out the overlapping and non-differentiation effects of BMSCs in the present vivo study, further research is needed. Of note, the application of BMSCs is beneficial to the treatment and prevention of endotoxemia in ALF, and the intestinal differentiation of BMSCs contributed to the repair of the intestinal mucosal barrier.

In summary, we showed the therapeutic potential of BMSCs in ALF by repairing the intestinal epithelial barrier and preventing endotoxemia, and the PI3K/AKT/mTOR signaling pathway was involved in the intestinal differentiation of BMSCs. These data not only alarm us about the role of intestinal endotoxemia in ALF but also inspire us to build an effective cell therapy in the treatment of ALF.
